# Post-partum hemorrhage: a multidisciplinary approach to ‘the golden hour’ quantum leadership and communication

**DOI:** 10.2144/fsoa-2023-0076

**Published:** 2024-05-15

**Authors:** Georges Yared, Jihad al Hasssan, Charlotte Al Hajjar, Kariman Ghazal

**Affiliations:** 1 Obstetrics & Gynecology Department, Rafik Hariri Hospital University Medical Center, Beirut, Lebanon; 2 Obstetrics & Gynecology Department, Lebanese American University, Beirut, Lebanon; 3 Obstetrics & Gynaecology Department, Lebanese University, Beirut, Lebanon; 4 Obstetrics & Gynaecology Department, Al Zahraa Hospital University Medical Center, Beirut, Lebanon

**Keywords:** delivery, healthcare, medication, medwife, obstetric skills, postpartum hemorrhage, pregnancy, quantum, quantum leadership, simulation

## Abstract

**Background:** Quantum leadership in postpartum hemorrhage (PPH) simulation training remains underexplored. Quantum leaders excel in PPH's chaotic settings, differing from traditional leaders. **Aiming:** To assess the impact of simulation training on quantum leadership skills in maternity teams. **Research design:** A quasi-experimental approach. **Sample:** 60 maternity professionals from Rafik Hariri University Hospital, Lebanon. **Tools:** Structured interviews, observational checklists and a leadership skills scale. **Results:** Most participants had limited PPH simulation experience and showed an initial low proficiency. Significant improvements were evident, post training. **Conclusion:** Simulation training enhances quantum leadership in PPH management among maternity professionals.

## Plain language summary

We looked at how a new style of leadership, called ‘quantum leadership’, can help medical teams better manage a serious childbirth complication called postpartum hemorrhage (PPH). This leadership style is especially good in unexpected and chaotic situations like PPH. Our study took place in a special training lab at Rafik Hariri university hospital in Lebanon, involving 60 medical professionals. We found that many had not been trained this way before and had less than 10 years of experience. Their starting skills and confidence were low. However, they showed significant improvement after our training. This shows the importance of this kind of training for childbirth care professionals.

## Abstract (plain language summary)

Simulation-based training is pivotal in enhancing quantum leadership skills, especially in managing PPH.

Quantum leadership fosters a unified vision between the leader and the team, promoting teamwork and better patient care:
  - Quantum seeing: situational awareness through risk assessment.
  - Quantum knowing: early recognition of PPH.
  - Quantum feeling: emphasizing positive aspects and early engagement of specialists.
  - Quantum thinking: quick adaptation to new environments.
  - Quantum acting: rapid response within the ‘golden hour’ for maximum survival chances.
  - Quantum being: strong team communication, collaboration, and cooperation.
  - Quantum trusting: monitoring, evaluation, and continual reassessment.

The transformative potential of quantum leadership in healthcare is particularly evident in managing PPH.

Public awareness is key to maximizing access to and effectiveness of primary care.

Social networks can play a role in driving health behavior changes.

Incorporating advanced care planning into primary care can reduce hospital admissions and elevate patient satisfaction.

Primary healthcare should be prioritized with active community participation.

Sexual and reproductive health services need integration in disaster and humanitarian contexts.

Early research integration in humanitarian initiatives helps in pinpointing and addressing service gaps in less accessible populations.

Strengthened community outreach is crucial for enhancing awareness about available healthcare services and emphasizing the importance of preventive care.

Quantum leadership is rooted in the principles of quantum physics and views organizations as complex, interconnected systems rather than linear entities. Conventional leadership, on the other hand, often sees organizations in a hierarchical and systematic manner [1,2].

## Perspective on chaos

Quantum leaders thrive at the edge of disorder and uncertainty, constantly adapting and looking for innovative solutions. Traditional leaders typically aim for stability and predictability [^3-6^].

## Skills and approach

Quantum seeing allows leaders to envision multiple scenarios and adapt their perceptions based on changed purposes. Quantum knowing: combines rational and intuitive insights, enabling more mindful decisions. Quantum feeling: emphasizes positive, motivating emotions, focusing on solutions rather than problems. Quantum thinking: embraces paradoxical solutions and encourages out-of-the-box problem-solving. Quantum acting: prioritizes accountability, rapid responses and the efficient use of resources. Quantum being: emphasizes connection, communication, collaboration and cooperation, fostering a unified vision. Quantum trusting: ensures consistent monitoring and evaluation of performance [^7-9^].

## Role of leaders

Quantum leadership values the energy and interconnectedness between leaders and team members, prioritizing shared visions and goals. Traditional leadership often places the leader in a directive, authoritative position [1,9].

## Training and learning

Quantum leadership emphasizes simulation-based training, allowing teams to practice complex procedures in a controlled environment. This approach can lead to significant improvements in outcomes, such as a reduction in maternal deaths in the case of PPH management [1,2,5,10].

## Change management

Quantum leaders promote cooperation, collaboration and negotiation when implementing change, rather than an authoritative approach. They recognize the shared responsibility of all stakeholders in improving outcomes [9,11].

## Outcome-driven approach

Quantum leadership focuses on value-driven outcomes that matter to patients, using tools like the 4Rs (readiness, preparedness, response, reporting) to ensure holistic care. In essence, while conventional leadership aims for balance, predictability and a top-down approach, quantum leadership celebrates uncertainty, interconnectedness and a collective vision to drive change and innovation [1,5,6,10].

## Objective

This study evaluates the impact of simulation-based training on improving the quantum leadership skills of a maternity team in managing postpartum hemorrhage (PPH) at Rafik Hariri University governmental hospital, Lebanon. Quantum leaders operate at the edge of chaos, and are essential in managing complex situations like PPH.

## Materials & methods

### Study design

The study is an experimental design examining the impact of simulation-based training on quantum leadership skills among frontline maternity teams, including midwives, residents and obstetricians. The goal is to improve their response in emergency care, specifically in the 4Rs care bundle of post partum hemorrhage PPH management. Data collection will be done using a self-constructed questionnaire, both pre and post-training. This questionnaire is inspired by the Gallup Strengths finder, focusing on strengths in line with the quantum leadership assessment. It will measure seven quantum leadership skills using a 5-point Likert scale. Scores below 50% indicate low quantum leadership skill, between 50 and 75% indicate moderate skills, and above 75% indicate high skills.

### Course description

The obstetrics simulation training course is designed to provide hands-on experience for medical professionals in the field of obstetrics. Using advanced simulation techniques, including mannequins, the course aims to replicate real-life scenarios, ensuring trainees are well-equipped to handle a variety of situations.

### Course contents

- **Introduction to obstetrics simulation:** Understanding the need and importance of simulation in medical training;
- **Basic obstetric procedures:** Hands-on training with mannequins, focusing on standard procedures and techniques;
- **Advanced procedures:** Dive deep into complex situations, such as C-sections, with an emphasis on the B-Lynch technique;
- **Emergency scenarios:** Training on handling emergency situations, understanding quick decision-making and prioritizing patient safety;
- **Balloon tamponade in normal delivery:** Detailed sessions on employing balloon tamponade during normal deliveries;
- **Feedback and continuous improvement:** Interactive sessions where trainees can discuss their experiences, learn from mistakes and understand best practices;
- **Trial runs:** Using mannequins to simulate real-life scenarios, allowing trainees to test their skills in a controlled environment.

#### Tutors

Our team of tutors comprises experienced obstetricians and gynecologists, along with experts in medical simulation. They bring a wealth of knowledge and practical experience to ensure trainees receive top-tier education.

#### Duration

The course spans over a period of 2 weeks, with daily sessions lasting approximately 6 h. This intensive format ensures thorough coverage of all topics while providing ample hands-on practice for all trainees.

#### Additional notes

While the primary mode of training involves mannequins, we prioritize safety and ensure that no real patients are involved in the simulation exercises.

Participants' demographic details will also be captured. The questionnaires will be distributed online before and after the training, and the collected data will be analyzed using the SPSS software and *T*-test statistical methods.

##### General analysis

Multiple linear leadership styles were used to improve PPH management with no or limited benefits specifically on maternal outcomes since PPH is still the leading cause of maternal deaths [12].

According to John Maxwell, *“A leader knows the way, goes the way and shows the way.”* While vision without action is a dream and action without vision is a waste of time; vision with action can achieve the goals [9].

Lewin and Lippitt compared three styles of leadership in 1939: autocratic, democratic and laissez-faire [2,13].

Over the years many styles have emerged within the social vocabulary, but all of them can find their roots within these three styles of leadership like bureaucratic, transformational, transactional and servant leadership styles [6].

The autocratic leader retains power (classical approach) and he (more than she) is the sole decision-making authority and does not consult employees for any input. While this style is effective in emergencies, it is less efficient when it comes to complex and chaotic situations like PPH. Since subordinates are not involved in decision making they are like slaves, and the environment is like a prison. The democratic and bureaucratic styles are slow-paced and do not fit the timeliness and agility of response in life-threatening situations. Transformational and transactional leadership styles are effective in change management and are less effective when high performance and speed of action are needed like the ‘golden hour’ in PPH management [^14-16^]. These leadership styles have a vertical gravitational orientation with hierarchical structures, and process-driven action and are effective in traditional predictable and preventable situations. Whereas the quantum approach has multifocal characteristics, based on connectedness, communication, collaboration, cooperation and shared vision, its structure is nonlinear like heterarchy. Warren McCulloch was the first to describe the concept of heterarchy [^16-19^].

Heterarchy is a dynamic hierarchy including a mixture of levels. There is a simultaneous interaction among levels, and it gives rise to the re-organization of the structure [3,18].

This wholeness seals the discussion of the interaction between parts and a whole [10]. It is a value (outcome) driven action and works best in chaotic and complex situations which are unpredictable and unpreventable like PPH [20].

A continuation of multiple chaotic shock waves and fluctuations of disasters in the foundations Lebanon has faced during the past years [^21-23^]. In 1948, tens of thousands of refugees came to Lebanon from Palestine to live in temporary camps set up and overseen by the United Nations Relief and Welfare Agency (UNRWA) protection at UNRWA in 2016.

Between 2011 and 2013, the Lebanese population increased by 30% due to the influx of Syrian refugees [24]. The massive impact of the COVID-19 pandemic further hastened the collapse of the Lebanese healthcare system [25] and pushed a large proportion of its population below the poverty line [26]. An unprecedented Lebanese economic crisis in 2020 limited health expenditure, primary healthcare is considered the core of the health system and is based on the principles of ‘justice, equality and rational use of resources’. Nevertheless, the reality differs from that axiom, given the Lebanese healthcare system is oriented toward curative care.

The ministry of public healthcare (MOPH) only allocates 5% of its budget to preventive primary health centers PHC services.

The Lebanese healthcare system relies primarily on the private sector which represents 80% of hospitals and 68% of primary health centers (PHC) owned by non-governmental organizations. In Lebanon we have a fragmented health system highly competitive based on isolation and rivalry, mostly relying on the private sector focused on volume and profit [27]. The humanitarian health response has been fragmented and often inefficient [22,23].

Factors include a lack of awareness among the populations residing in the most underserved areas of Lebanon of the availability of supported services and challenges in ensuring cost control in a dominant privatized health system operating outside the MOPH network [28]. PHC centers do not report standardized national indicators, but plans are underway to establish those indicators. Indicators of success are only available for the 17 publicly owned centers accredited in 2011 by the Canadian [29]. Availability of information is the major concern in measuring outcomes of care. Without systematic collection and reporting of certain outcome information to central review bodies, it is difficult to monitor and improve this aspect of quality of care. Instead, structure and process measures have to be used to measure the quality of care PHC.

Limited information on the prevalence and severity of health conditions such as NCDs and mental health issues across target groups, lack of information on utilization rates of hospitals and response capacity in terms of quality of health services, availability of medications and lack of data on how social determinants of health (e.g. education, shelter housing) are linked to the health status. The Health intervention was effective in reaching its target population, diabetic and hypertensive patients living in rural areas and Palestinian refugees [24].

## Results

### Maternal & newborn health

Regarding maternal and newborn health, it was reported that normal deliveries were available/possible in 9.1% of facilities. The statement, *“in 9.1% of facilities, it was noted that normal births were available or practicable in terms of mother and neonatal health”* implies that out of all the facilities studied, only 9.1% of them provided conditions where normal births (those without interventions like cesarean sections) were feasible or practical while also ensuring the health and safety of both the mother and the neonate (newborn). In other words, in these 9.1% of facilities, mothers could expect to have a natural birth without compromising their health or the health of their babies [29]. Basic Emergency Essential Obstetric Care (BEmOC), skilled care during childbirth, and vaccination during pregnancy were available in 12.7, 21.8 and 23.6% of facilities, respectively. Other maternal and newborn health services such as postpartum care, antenatal care, and tetanus shots, are available in 50–60% of the facilities (45.5%) [29].

Despite the integral role of PHC in health systems, the World Health Report [30] indicated that countries *“are not performing as well as they could and as they should”* when it comes to PHC.

Access to secondary care: access to secondary care in Lebanon has no limits. Any pregnant patient can choose to use the services at any level of care without any referral. One can choose to go to a specialty obstetrician without passing through a midwife and choose to perform certain lab tests according to his request as long as he pays for the services immediately. However, referrals are required in health centers and in case a third-party guarantor is involved [29].

Autonomy of public hospitals in Lebanon until 1996, public hospitals in Lebanon operated a budgetary unit of the MOPH with central planning and central decision-making. Because of the dismal results of this centralized model, the Lebanese government GOL decided to give autonomy to the public hospitals, and law 544/96 on ‘public hospital autonomy’ was passed in 1996; it allowed the creation of ‘public institutions’ to manage public hospitals. This law was based on the institutional model of 1972, decree no 4.517/72 on ‘General Regulation of Public Institutions’. Amendment law 602/97 and four implementing decrees followed law 544/96. This law and the following amendment and decrees did not give any significant autonomy (financial or managerial) to the public hospitals, which remained under the tight control of the Ministry of health MOH Ministry of public health and Ministry of Finance (MOF). In terms of hospitals, though the public sector is the main payer for hospital care, the private sector dominates hospital service provision. Of the 165 hospitals in Lebanon, 82% are privately owned and managed by physicians or by charitable organizations. Public hospitals operate under a semi-autonomous model: the hospital boards are composed of various stakeholders so they have a certain degree of autonomy [13,21,31,32].

The World Health Organization [33,34] released a consensus statement and full strategy paper on ending preventable maternal mortality (EPMM). The EPMM target for reducing the global maternal mortality ratio (MMR) by 2030 was adopted as sustainable development goal SDG target 3.1: reduce global MMR to less than 70 per 100,000 live births by 2030. While maternal mortality declined by 38% between 2000 and 2017 worldwide, in Lebanon it didn't change and is still around 29 deaths per year. Frequently, those who are most supportive of change, or least resistant to it, are those who have confidence in their *“ability to win”* [35]. PPH management remains *“weak, fragmented and poorly targeted.”* The organized limited health institutions failed to meet the demands of those most in need who are usually too poor or geographically or socially remote to benefit from such facilities and lack awareness among the populations residing in the most underserved areas. The health services often created were in isolation, neglecting other health institutions and stressed curative services with insufficient priority to preventive, promotion and rehabilitative care [36]. Lebanon rarely has adequate outcome measures, such as complication rates, disease-specific mortality rates by the hospital, or functional states of patients after treatment to assess the quality of care MOF.

**Figure 1.**
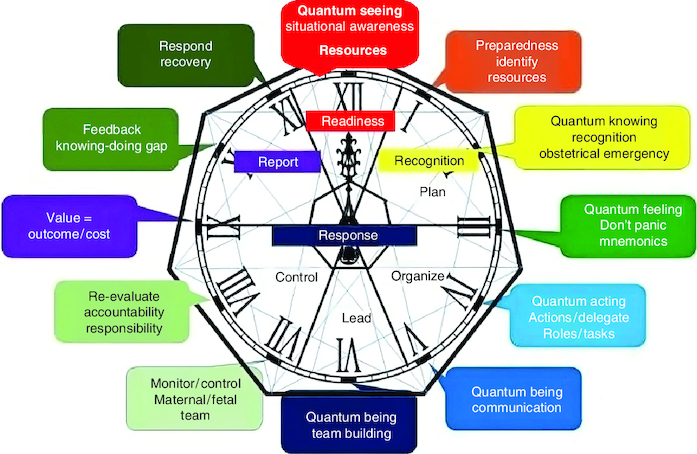
The quantum OB-wheel.

There are seven quantum skills: the order of the skills was selected by the order of the 4Rs care bundle (Figure 1) [2].

#### Quantum seeing

The ability to see deliberately [9]. A quantum leader has a clear vision, this is situational awareness through the assessment of risk factors of PPH, prevention, readiness and preparedness. Antenatal correction of anemia and identification of hemostatic disorders. In addition to systematic active management of the third stage of labor and manual removal of the placenta after 30–60 min. Conducting multidisciplinary simulation training at least once a year to improve the evaluation of blood loss. A good team must have as its priority meeting the patient's needs. A team with a patient-centered focus will consider and respect the patient's values and preferences when making care decisions.

#### Quantum knowing

The ability to know intuitively [6]. This is early recognition of PPH (blood loss >1 l/24 h or >500 cc after delivery) or severe PPH when blood loss >2 l. Identified as a soft, poorly contracted (boggy) uterus. MROP should be carried out under anesthesia if the placenta has not been expelled 30 min after active management of 3rd stage labor or 60 min [24,34].

Check if the placenta has been delivered and if is it complete. Prophylactic oxytocin reduces the risk of PPH by 60%. If oxytocin and bimanual uterine massage fail to sustain uterine contractions and control hemorrhage, consider transferring the patient to the hospital. A quantum leader knows how to integrate the rational and the intuitive. Active management of the 3rd stage is a systematic action. The positive connection between team members and the leader produces invisible energy. Most PPH has no risk factors, normal physiologic changes in pregnancy can mask the clinical signs and symptoms of PPH like air hunger, blood pressure (diastolic will rise and systolic will drop, severe if systolic <100 mmHg), pulse rate (severe if >30) and mental status. Labs Hb and Ht will drop only after starting the transfusion (severe if Hb <70).

#### Quantum feeling

The ability to feel active [9]. Focus on positive aspects instead of the problems: ‘don't panic’ and call for help. Early involvement of anesthesiologist. Identify the availability of drugs and blood products. Evaluation of blood loss/measure blood loss to estimated blood loss.

#### Quantum thinking

*“Too little is done too late”* this is the beginning of the response phase, the ability to think adversely [6]. Quantum leaders quickly adapt to the new chaotic environments and adopt out-of-the-box new solutions. Follow the 4Ts mnemonic tone 70%, trauma 20%, tissue 10%, and clotting disorder <1%. Follow the NATA consensus or FIGO algorithm.

#### Quantum acting

The ability to act accountable with a fast response [37]. The golden hour is the time from the onset of PPH during which resuscitation needs to begin for the patient to receive maximum benefit. The longer it takes between the onset of shock and resuscitation, the lower the chance of patient survival. Get the emergency trolley, start medications and prepare blood and blood products. In the ‘quantum acting’ simulation, while I approach the topic beyond a strictly obstetric perspective, I have ensured accuracy by adhering to both my personal guidelines and the established protocols of the hospital.

Give oxygen 100% with a face mask, empty the bladder, start uterine massage (abdominal or bimanual), keep the patient warm, insert two large bore cannulas and infuse 2 l of crystalloids, obtain labs (type and cross, CBC and coagulation factors) at a rate 6:4:1, or 4:4:1 of RBCs, FFP, platelets, respectively, prepare massive transfusion pack (consider the need to defrost) and fibrinogen if EBL >1.5 l, remove intra-uterine clots or tissues, repair laceration under anesthesia.

▪ Medications: oxytocin, 10–20 IU in 1 l, run 500 cc in 10 min then 250/h, maintenance dose, 40 IU in 500 ccs normal saline/4 h;
▪ Misoprostol: prevention, 600 mcg sublingual/rectal. The sublingual route is faster. Treatment 800 mcg not to exceed 1000 mcg;
▪ Methylergonovine 0.2 IU Q2–4 h. Ergometrine 0.5 mg IM;
▪ Carboprost: PGF2alpha, Hemabat, 0.25 mg q15 min, eight doses maximum, consider surgical treatment after the second dose.

Tranexamic acid: Exaxyl, 1 g slow IV Bolus, followed by 1 g after 4 h. Mechanical tamponade: Bakri balloon. For the sake of rapid response during a C-section, we begin with the B-Lynch techniques. As for balloon tamponade, we employ it during a normal delivery.

Surgical: embolization, internal iliac arteries ligation, or hysterectomy. Consider intraoperative cell salvage.

#### Quantum being

The ability to be in connection through communication, collaboration and cooperation without any restriction [9]. Teamwork is the ability to work together toward a common vision, and goals. The quantum leaders and the followers are completely bound together: the same vision, goals, knowledge and emotions. Delegate roles and tasks appropriately taking into consideration the competence and commitment of your team members. Use closed loop, eye contact and names. Messages should be well formulated, addressed, delivered, heard and understood. Decrease the risk of noise and miscommunication.

#### Quantum trusting

The ability to monitor, control, verify and evaluate [6]. Evaluate the performance of your team, monitor your patient and any change in the severity, and if so reconsider restarting the quantum Ob-wheel. Regularly check blood pressure, pulse rate, pulse pressure, respiratory rate and mentation.

The outcome is value-driven, where value is defined as *“the health outcome achieved that matters to patients relative to the cost of achieving that outcome.”* In the end, measure the outcome, and identify the gaps through immediate feedback.

Reporting using a standardized PPH chart. If the leaders use the seven quantum skills, they can see, know, feel, think, act and trust in ways that they can change for the better.

## Discussion

Improving public awareness about palliative care PC and its benefits can enhance access to PC, increase referrals to PC, and can help in identifying gaps in services provided. Social network interventions can promote health behavior change in consumers and can be used as a tool by organizations and policymakers to influence health behavior changes. Increasing knowledge and promoting awareness about PC can empower communities to participate in decision-making related to PC implementation considerations [38]. Advance care planning is an essential component of the PC. Evidence shows that advanced care planning is associated with fewer hospital admissions, improved patient and family satisfaction, and reduced healthcare costs. Public education on matters of road injury prevention, accident hazards and first-aid maneuvers, MOF [31,32].

Integrated care is often contrasted with fragmented care, and it is used synonymously with terms like coordinated care. Integration of care can take many forms [27,31,32].

We recommend primary healthcare should be given priority with the full participation of the community. It should serve as gate-keeping with adequate referral mechanisms for secondary and tertiary HC services. Sexual and reproductive health services are essential core services that need to be provided in disaster and humanitarian settings, as women and children can be highly affected in a crisis [29]. Concerning STIs, testing and counseling, those services are scarcely available. Safe identification and referral of gender-based violence (GBV) survivors are available in around 51% of the facilities, and psychological support and counseling in 41.8% [29].

### Focus on prevention

The importance of integrating research in the early stages of the humanitarian effort is to ensure that important gaps in hard-to-reach populations are identified and responded to [28].

### Limitations

While this study provides essential insights into the utilization of healthcare services for women of reproductive age and children, there are several limitations to consider:

- Service utilization: gaps were found in the use of health services like immunization, sexual and reproductive health (SRH), and non-communicable diseases (NCD) care. The study didn't fully explore all reasons for these gaps;
- Community outreach: the need for improved outreach is clear, but specific barriers to outreach remain undetermined;
- Resources and medication: the study highlights human resources and medication needs but doesn't detail current staffing or prescription practices;
- Generalizability: the adaptability of findings to other settings or regions wasn't thoroughly investigated.

Gaps in terms of utilization of healthcare services for women of reproductive age and children, particularly in immunization, SRH and NCD care, despite the support and availability of such services at the facility level. The study findings also suggest that a stronger community outreach effort is needed to improve awareness of the availability of services and knowledge about the importance of preventive care. Support to services provided must also ensure that adequate human resources are available and essential medication is proactively prescribed by healthcare providers to avoid preventable out-of-pocket spending. The findings of this study highlight the importance of integrating research in the early stages of the humanitarian effort to ensure that important gaps in hard-to-reach populations are identified and responded to [28].

## Conclusion

The emphasis on improving public awareness about palliative care is paramount for enhanced service accessibility and informed community participation in palliative care decision making. Integrated care, juxtaposed against fragmented care, emerges as a holistic approach to healthcare, necessitating coordination at various levels. The recommendation leans toward prioritizing primary healthcare, ensuring community involvement, and emphasizing the provision of essential services like sexual and reproductive health in crisis situations. Furthermore, the evident scarcity in services for STIs and GBV survivors underscores the need for improved facilities and psychological support. The proactive integration of research during early humanitarian interventions is crucial to address gaps in care, especially in hard-to-reach populations. This discussion underscores the vital role of prevention and a collective approach to address diverse health challenges.
